# Decoding substance use disorder severity from clinical notes using a large language model

**DOI:** 10.1038/s44184-024-00114-6

**Published:** 2025-02-07

**Authors:** Maria Mahbub, Gregory M. Dams, Sudarshan Srinivasan, Caitlin Rizy, Ioana Danciu, Jodie Trafton, Kathryn Knight

**Affiliations:** 1 Oak Ridge National Laboratory, Oak Ridge, TN USA; 2 Program Evaluation and Resource Center, Office of Mental Health and Office of Suicide Prevention, Veterans Health Administration, Department of Veterans Affairs, Menlo Park, CA USA

**Keywords:** Health care, Public health

## Abstract

Substance use disorder (SUD) poses a major concern due to its detrimental effects on health and society. SUD identification and treatment depend on a variety of factors such as severity, co-determinants (e.g., withdrawal symptoms), and social determinants of health. Existing diagnostic coding systems used by insurance providers, like the International Classification of Diseases (ICD-10), lack granularity for certain diagnoses, but American clinicians will add this granularity (as that found within the Diagnostic and Statistical Manual of Mental Disorders classification or DSM-5) as supplemental unstructured text in clinical notes. Traditional natural language processing (NLP) methods face limitations in accurately parsing such diverse clinical language. Large language models (LLMs) offer promise in overcoming these challenges by adapting to diverse language patterns. This study investigates the application of LLMs for extracting severity-related information for various SUD diagnoses from clinical notes. We propose a workflow employing zero-shot learning of LLMs with carefully crafted prompts and post-processing techniques. Through experimentation with Flan-T5, an open-source LLM, we demonstrate its superior recall compared to the rule-based approach. Focusing on 11 categories of SUD diagnoses, we show the effectiveness of LLMs in extracting severity information, contributing to improved risk assessment and treatment planning for SUD patients.

## Introduction

Substance use disorder (SUD) is a multidimensional health problem growing at a national scale. The complexity of SUD assessment and treatment is based on several factors, including symptom expression, patterns of substance use over time, co-morbidities, and other social determinants of health impacting clinical presentation and treatment outcomes. Despite this complexity, the codes available for identifying patients with SUD, such as the International Classification of Diseases (ICD-10)^1^, in clinical health records lack granularity for indicating these additional factors, which makes it difficult to automatically flag any patients who may present specific risk factors. In addition to the ICD-10 coding system, the Diagnostic and Statistical Manual of Mental Disorders classification (DSM-5)^2^ is also used to set criteria for various disorders as defined by the American medical community. However, the DSM-5 does not designate a unique numeric code for any of the conditions it lists (and therefore cannot easily enter into a structured electronic healthcare record like an ICD-10 code can), nor is there a one-to-one equivalency for a DSM-5 diagnosis and an ICD-10 code diagnosis.

When clinicians use DSM-5 to diagnose their patients and ICD-10 to codify this information, it is not uncommon that the more granular DSM-5 SUD diagnostic information is recorded by a clinician in an unstructured note. To date, many traditional natural language processing (NLP) efforts have been tested and implemented to identify additional patient risk factors in unstructured clinical notes, with varying degrees of success^3,4^. However, the high variability in language found within clinical notes imposes significant limits on the accuracy of traditional techniques that rely on parsing rules to detect text patterns. Clinician typos, neologisms, abbreviations, and other linguistic variations hinder the effectiveness of these methods. Deep learning techniques, on the contrary, have demonstrated impressive efficacy in extracting such information from complex unstructured clinical notes^4,5^. Nevertheless, as information extraction is a supervised NLP task, the requirement for large-scale annotated datasets of high quality for training these models to reach their full potential poses an inevitable bottleneck in novel real-world applications. Recently, large language models (LLMs) have emerged as a promising solution to this challenge, particularly due to their ability to “learn” and adapt to diverse language patterns without the need for additional model training^6^.

LLMs are deep learning models in NLP equipped with millions or billions of parameters. They have revolutionized NLP by demonstrating a remarkable ability to interpret and generate human-like text across a wide range of domains^6,7^. Although primarily trained on open-source, non-domain-specific texts, some studies have underscored their effectiveness when applied to clinical notes^8–14^. Unlike traditional deep learning models that require extensive domain-specific training data to achieve high performance, LLMs leverage their vast pre-training on diverse text to interpret context, make inferences, and generate relevant responses. Two techniques, namely zero-shot learning (ZSL) and few-shot learning (FSL), play pivotal roles in the context of LLMs. In both methods, the LLM performs a novel task on unseen data guided by a user-defined task description (prompt). The crucial distinction between ZSL and FSL is the extent of exposure the LLM receives during inference. ZSL entails no exposure, while FSL involves exposure to a limited number of examples. This is particularly valuable in the clinical domain, where acquiring expert-annotated data is both costly and time-consuming.

Recent studies have explored the application of LLMs in the clinical domain^8–11^. Alsentzer et al.^12^ used ZSL with the Flan-T5 model to extract postpartum hemorrhage (PPH) concepts from obstetric discharge summaries for interpretable phenotypes, achieving a high positive predictive value and identifying 45% more patients with PPH than claims codes. Agrawal et al.^13^ used ZSL and FSL with GPT-3 based InstructGPT model^15^ to perform five tasks in the clinical domain: clinical sense disambiguation, biomedical evidence extraction, co-reference resolution, medication status extraction, and medication attribute extraction. Their findings demonstrated that GPT-3 achieves better performance than established baselines using guided handcrafted prompts. *HealthPrompt*^16^ is a ZSL paradigm introduced to classify new classes without prior training data. The authors utilized ZSL to enable prompt-based learning by adjusting task definitions via 2 types of prompt templates: cloze prompts and prefix prompts. Using various pre-trained models and custom prompt templates, they demonstrated improved performance, particularly notable with ClinicalBERT^17^, which was trained on clinical texts. In another study^14^, custom prompts were used to extract information from breast cancer pathology and ultrasound reports. They developed 12 prompts for extracting and standardizing data, finding that pre-trained language models, such as those from OpenAI, enabled efficient and cost-effective information extraction.

Numerous studies have focused on extracting targeted information related to substance use, employing rule-based techniques or a combination of rule-based and machine-learning methods across various substances, including opioids^3,18,19^, alcohol, tobacco, and drugs^20–24^, and cannabis^25,26^. These studies underscore the need for traditional manual annotation. Recent research, exemplified by refs. ^9,27,28^, has employed ZSL and FSL prompting techniques within LLMs such as GPT-3.5, GPT-4, and Flan-T5 to extract SDoH information from clinical notes, marking a shift in approach towards data annotation challenges.

In this study, we investigate the utilization of LLMs to address the challenge of limited annotated datasets in a new clinical application—specifically, extracting information on the diagnostic severity specifiers of various SUD diagnoses from clinical notes. Our proposed workflow involves performing zero-shot learning (ZSL) of LLMs by carefully crafting prompts and post-processing the generated text to extract DSM-5 SUD diagnoses with their diagnostic specifiers containing severity-related information (i.e., severity level and remission status). In a zero-shot learning scenario, the LLM is prompted to execute a specific task via an input prompt, generating text in response, which serves as the output. For instance, when provided with the prompt: *Extract the reference to alcohol use disorder diagnosis with surrounding information relevant to it from the diagnoses section in the following note. If you can’t find the answer, please respond “unanswerable*”*. Note:* <*clinical note text*>. Then, the LLM produces the extracted text—*severe etoh use d/o*—from the clinical note. Leveraging Flan-T5^29^, an open-source LLM, we demonstrate through experimentation and comparative analysis that the LLM achieves better recall compared to rule-based regular expressions, highlighting its efficacy in this downstream task without requiring additional training. For evaluation, we use a set of 577 notes annotated by a subject-matter expert (SME) who is a Licensed Clinical Psychologist. We focus on 11 categories of SUD diagnoses: alcohol; opioids; cannabis; sedatives, hypnotics/anxiolytics; cocaine; amphetamines; caffeine; hallucinogens; nicotine; inhalants; and other psychoactive substances. To the best of our knowledge, no previous study has employed LLMs to extract information on severity specifiers of SUD diagnoses across 11 SUD categories. This study is an initial step in our larger objective to devise an efficient approach to use LLMs to extract an array of nuanced information on SUD diagnoses, including severity specifiers, withdrawal symptoms, and social determinants of health, thereby contributing significantly to risk assessment, treatment planning, patient safety, recovery, and overall well-being.

## Methods

**Fig. 1:**
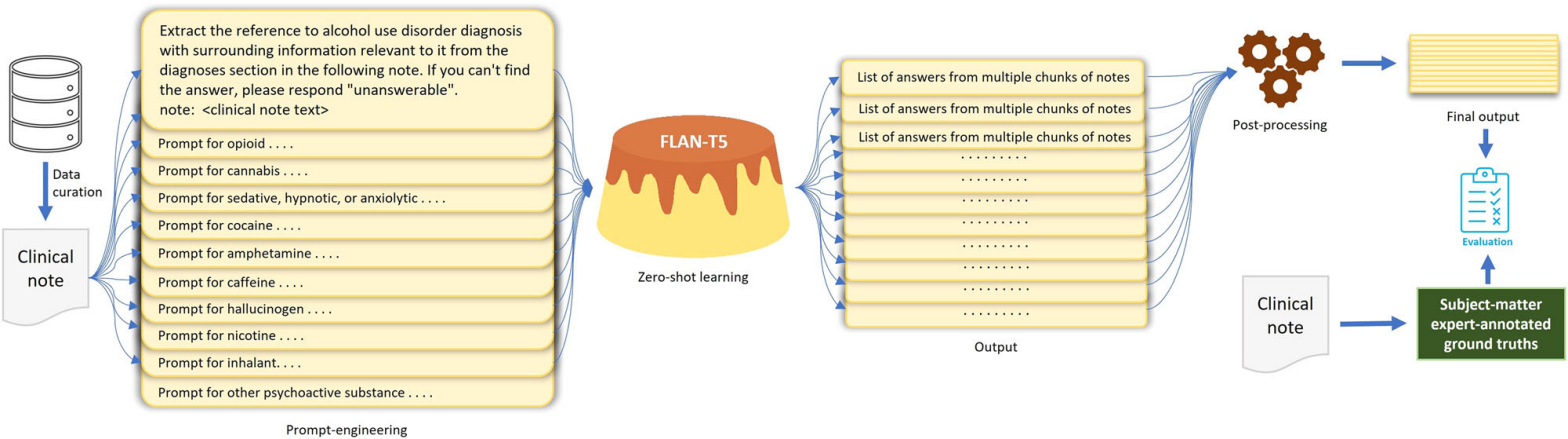
Overall workflow of the ZSL approach with a large language model FLan-T5. The steps in the workflow include data curation, data annotation, prompt engineering, zero-shot learning, post-processing of the model-generated output text, and performance evaluation.

In this section, we describe the task and concepts of interest, our dataset, the ZSL approach, and the experimental setup for using LLMs to extract information on DSM-5 SUD diagnoses with their respective diagnostic severity specifiers. We also briefly outline performance evaluation metrics. Figure 1 shows the overall workflow of this study, including data curation, annotation, information extraction using an LLM, post-processing, and evaluation.

### Dataset

For the study, we used a random set of 577 fully identified clinical notes belonging to 574 unique patients, curated from the United States Department of Veterans Affairs (VA) Corporate Data Warehouse. While sampling, we ensured that the selected notes were associated with any of the following ICD-10 codes in the structured data—*F*10, *F*11, *F*12, *F*13, *F*14, *F*15, *F*16, *F*17, *F*18, *F*19—to increase their chance of containing information on SUD. Additionally, we augmented this selection with the following structured data criteria: admittance to a SUD clinic, presence of a CPT code of interest, but not any notes related to group therapy or drug test results. The notes were taken during patient visits occurring between October 2015 and November 2023. The notes vary in length from 11 to 10719 tokens, with an average of 1043 tokens and a median of 423 tokens. The types of notes comprise 79 varieties, including but not limited to mental health, suicide prevention, SUD treatment programs, education, psychiatry, administrative, psychology, and nursing.

For the concept of interest, we aim to extract severity specifiers along with mentions of SUD as a single continuous string, as they appear in the clinical note. For example, “meets criteria for substance use disorder: cocaine [] mild (2–3); [] moderate (4-5); [x] severe (6 or more)”, “mild cocaine use disorder”, or “inhalant use disorder—in sustained remission”. Our rationale is that a severity specifier for a diagnosis holds clinical significance only when it is associated with the corresponding diagnosis. Another factor to consider is that for a mention of SUD, such as “moderate alcohol use disorder”, to qualify as a diagnosis, there must be an indication in the note that the attending clinician has identified it as a “diagnosis”, rather than merely describing a past or present problem. In cases where there is no mention of severity, it is clinically equivalent to having an unspecified severity, and therefore, only the mentions of the diagnoses will be extracted. For example, “inhalant (nitrous oxide) use d/o”. Documentation of SUD severity specifiers in the clinical notes constitutes only explicit mentions of severity, for example, “mild” in “cannabis/alcohol/opioid use disorder: mild”, “moderate, in remission” in “other hallucinogen use disorder (mdma/ecstasy), moderate, in remission”, “continuous use” in “alcohol use d/o, continuous use”, or “severe” in “meets criteria for substance use disorder: cocaine [] mild (2–3); [] moderate (4–5); [x] severe (6 or more)”. The absence of such explicit severity specifiers indicates “unspecified” severity. Phrases such as the frequency of use or effects of use on a patient’s health or personal life are considered insufficient information to determine a severity specifier and not included in the SUD diagnoses statements by clinicians. Note that because our task is to extract the clinician-assessed severity of the substance use disorder diagnoses, we did not consider any negated or historical mentions during annotation, apart from the cases where the historical mention is actively diagnosed by the clinician and therefore is still being assessed and treated, for example, the historical mention “in remission” in “cannabis use disorder, in remission”.

An SME manually annotated each note to establish ground truths for diagnoses and their respective severity specifiers across 11 categories of SUD diagnoses. Observations reveal that the information about the severity level of diagnosed SUD is usually found in the section(s) of the note that mentions the current diagnoses during that patient visit. We used this prior knowledge to build the prompts for the LLM later in the study. During our post-annotation exploratory data analysis, we noted a highly imbalanced ratio between notes containing and those lacking information for each of the SUD categories, ranging from a minimum of 3% to a maximum of 33%, with an average of 10%, as anticipated. Due to the privacy policies of the VA, exact numbers regarding the distribution of certain SUD categories cannot be shared, as they fall below the threshold established in the IRB agreement between ORNL and the VA. Nevertheless, in Section “Results”, we present the normalized count for each SUD category within the test set to assess the impact of this imbalanced data distribution on performance. Namely, the differential representation in SUD categories is consistent with variable SUD prevalence of patients using VA^30,31^. Equal distribution of note selection using ICD-10 substance categories was impractical for three reasons: notes selected based on SUD ICD-10 codes do not necessarily contain respective SUD diagnoses text, amphetamine and caffeine substance categories can use the same ICD-10 codes and thus could not be parsed a priori, and comorbidity of multiple SUDs occurs in about 24.4% of patients using VA with at least one SUD (VA, internal data) meaning that equal extraction of notes by ICD-10 codes would not equate to equal representation of SUD categories represented within note text. This challenge of targeted selection of notes with appropriate use of disorder information highlights the complexity of the task: if targeted disorder information were easily identified in the notes based on structured clinical data, this work would not be as complex and of significance.

Ethics: This project was conducted as a national quality improvement effort to improve care for veterans with substance use being treated in the VA. Models were designed to be implemented into VA decision support systems, and are not expected to be generalizable or valid for application outside of notes from the VA Computerized Patient Record System. As such, this work is considered non-research by VA (as per ProgramGuide-1200-21-VHA-Operations-Activities.pdf (va.gov)). However, Oak Ridge National Laboratory (ORNL) required additional oversight of this VA clinical quality improvement project as local standard practice for all uses of patient medical record data within their institution, with approval of the project by the ORNL IRB.

### Zero-shot learning using a large language model

In light of the high performance in clinical concept extraction from unseen text passages without further training demonstrated in recent studies^9,12^, we opted for the Google Flan-T5 models^29^ for our experiments. Flan-T5 is a family of large language models with encoder-decoder architecture, which was instruction-finetuned from Google T5 models^32^ on 473 NLP datasets for 1836 NLP tasks utilizing a diverse range of instruction templates. There are five models in the Flan-T5 family: Flan-T5-XXL, Flan-T5-XL, Flan-T5-Large, Flan-T5-Base, and Flan-T5-Small with 11B, 3B, 780M, 250M, and 80M parameters, respectively. In the context of zero-shot learning, the Flan-T5 model takes a prompt outlining the task as input and generates text as output in response to the prompt (Fig. 1). In this study, we leveraged Flan-T5 models to extract text indicating any substance use diagnosis severity specifiers from clinical notes using zero-shot learning. To develop prompts for the task, we used a small development set containing 57 randomly selected clinical notes of 10% of the patients within the dataset. The remainder of the clinical notes were considered as the test set. Note that there is no patient overlap between the development and test sets to avoid data leakage.

For prompt engineering, we drew inspiration from prior studies performing inference via zero-shot learning using Flan-T5 for clinical concept extraction^12,14^. In our use case, we found that the model often fails when presented with straightforward prompts, such as, “Extract the severity level of alcohol use disorder diagnosis in the following note. Note: <report text>”. To expedite progress, we employed a trial-and-error strategy based on prior knowledge acquired during data annotation. Specifically, our prompts are based on observing that the SUD severity specifier tends to be located adjacent to wherever the diagnosis is written in the note. Beginning with “alcohol use disorder”, we refined the prompt up to 10 iterations. Subsequently, we performed minimal prompt adjustments for the remaining SUD categories. We provided the developed prompts for all SUD categories in our GitHub repository (available at: https://github.com/mmahbub/SUDSeverity-Extract-LLM). Crucially, the location of a diagnosis in a clinical note is not based on any standardized note structure or format. Note structure widely varies based on providers, VA centers, locations, note types, and timeframes. Hence, we deferred to the LLM to locate the diagnoses within the note and extract information from it.

Clinical notes frequently surpass the maximum allowable sequence length of 512 tokens in the input text for Flan-T5 models. This challenge is compounded when prompts are added to the beginning of the clinical notes. While a potential strategy might be to read only the most relevant part of the note—e.g., the patient diagnosis–this is an impractical approach, as clinical notes lack any consistent structure. The Text Integration Utilities (TIU) used by the VA’s Veterans Health Information Systems and Technology Architecture (VistA) software package allows free-text documentation of patient encounters (including diagnosis, progress notes, and other relevant information), however, note attributes, hierarchy, and content vary depending on the type of patient care, note specialization (e.g., discharge summary, etc), clinician, facility, and specific TIU package deployment. This variety is spread throughout the 130+ different VistA systems in use across 1,250 VA healthcare facilities. As such, there is no practical or standard means to extract one “section” from a TIU note. To address this issue, we adopted a known technique in question-answering modeling: sliding window with a document stride^33^. In this technique, if the number of tokens in the input prompt is 50 and the number of tokens in the clinical notes exceeds 462, a sliding window with a document stride is applied to create chunks of the clinical note where each chunk consists of 462 tokens. We used 128 as the document stride based on previous studies^33^. Once the chunks are formed, the tokens from the prompt are prepended to each note chunk forming multiple input sequences of 512 tokens for the same note. Note that, the length of the clinical notes and the maximum sequence length also restrict the few-shot inference capability of the Flan-T5 models.

Considering that model-generated output text may contain irrelevant information as multiple chunks of the same note are presented to the model, we performed some post-processing steps for each SUD category from an operational point-of-view, as follows: (i) We removed answers that did not contain any of the phrases/substance names associated with the SUD category of interest. (ii) Additionally, we filtered out answers lacking any of the specified phrases representing “use disorder”, such as “dependence” or “use d/o”, following the substance names. The list of substance names is available in our GitHub repository along with the specified phrases denoting a use disorder. (iii) Furthermore, to increase the reliability of the generated text and reduce hallucination in the final output, we filtered out the answers if any of the common substrings of length five or higher (measured in characters) between the note and the answer do not satisfy the conditions explained in (i) and (ii). We chose a length of five for common substrings, determined by the shortest reference to substance use disorder in our dataset: “mj ud” (where mj and ud represent marijuana and use disorder, respectively). The post-processing steps were only performed on the answers that did not contain the string “unanswerable”. Any filtered answers were replaced with “unanswerable”. (iv) If the first three steps do not succeed in choosing one answer, we use a proxy ground truth for each SUD category to choose the final answer. Examples of proxy ground truths are—“cannabis use disorder/dependence” for “cannabis” and “opioid use disorder/dependence” for “opioid”. These proxies are chosen for simplicity based on our observation. We calculate similarity scores between the embeddings of proxy ground truth and candidate answers and pick the one with the highest score as the final answer. Note that the proxy ground truth is not chosen based on the actual ground truth but based only on information extracted by the LLM. We base our heuristic based on the assumption that answers containing information other than “unanswerable”, if they contain the correct information with correct SUD category, should be close to the proxy ground truth in the embedding space. We use the BERT encoder^33^ to generate the embeddings. For similarity metrics, we experiment with cosine similarity and Euclidean distance. Upon manual inspection, Euclidean distance proved more effective in selecting the best final answer, particularly regarding conciseness and relevance. While both metrics operate on BERT embeddings, which capture semantic relatedness, Euclidean distance may still offer advantages in this context as it considers not only the direction of the vectors (as cosine similarity does) but also their magnitude, which can help distinguish responses based on the overall size of the answers or subtle structural differences. In contrast, cosine similarity focuses purely on the angular relationship between vectors, emphasizing word usage patterns and semantic alignment without accounting for the length or scale of the responses. Additionally, Euclidean distance might be more robust when handling noise or outliers in the embeddings, which can disproportionately affect the angular measure in cosine similarity. For instance, when comparing candidate answers such as [“alcohol use disorder, severe” vs. “based on DSM-5 alcohol use disorder, severe, tobacco (cigarette) use disorder (moderate)”], Euclidean distance may better differentiate relevant content due to its sensitivity to differences in length or scale between the embeddings. Furthermore, to validate the effectiveness of this approach, we compare the best candidate answer selected via Euclidean distance with the one chosen using the relaxed match F1 score against the ground truth. We find that the Euclidean approach almost consistently selects answers that align with those chosen by the F1 score.

For our study, we conducted experiments with all variants of the Flan-T5 models and presented the evaluation scores for the best-performing one. We used the models from the HuggingFace^34^ model hub (available at: https://huggingface.co/docs/transformers/main/en/model_doc/flan-t5). For output text generation, we used the greedy decoding approach, setting the temperature to 1 and the maximum number of new tokens to 100. All our experiments were performed on a Linux virtual machine with two GRID V100-32C GPUs.

### Information extraction using regular expressions

We also performed information extraction using primitive rule-based regular expressions (RegEx). We created the rules following common patterns and using the phrases/substance names associated with the SUD categories, the terminology indicating a use disorder, and the phrases denoting their respective diagnostic specifiers. The complete set of rules is available in our GitHub repository. We compared the performance of the Flan-T5 model to the RegEx approach for each SUD category. Regular expressions serve as a fundamental benchmark since they, similar to the zero-shot learning of large language models, do not necessitate training.

### Evaluation

We measured the performance of the LLMs using two string matching criteria–strict and relaxed. As the names suggest, the strict match looks for character-by-character matches between the ground truth and the generated text while the relaxed match just looks for overlap. Based on these criteria, we used the F1, precision, and recall scores to assess the performance of the LLMs. In this scenario, True Positive denotes the number of tokens that are common between the ground truth and the generated answer, False Negative indicates the number of tokens present solely in the ground truth but absent in the generated answer, and False Positive represents the count of tokens exclusively found in the generated answer. The relaxed F1, precision, or recall scores per sample have a range from 0 to 1. Following^35^, for strict matching, we use binary values for precision and recall—either 0 or 1. Because the focus is solely on whether the extracted text exactly matches the ground truth, the F1 Score for strict matches is calculated as follows: (i) If both precision and recall are 1, the F1 Score is 1. (ii) If recall is 1 but precision is 0, the F1 Score is 0. (iii) If recall is 0, the F1 Score is always 0, regardless of precision. For “No SUD” cases, if there are no tokens in the generated answer and as such no false positives, then F1 would be 1. If there are some tokens in the generated answer (false positives), then F1 would be zero. As per a prior study^36^, we present the macro-averaged F1, precision, and recall scores, on the test set.

## Results

We report the performance of the best-performing Flan-T5 model, Flan-T5-XXL, in the zero-shot learning paradigm, specifically tailored to our task: extracting information for SUD diagnosis severity specifiers. The Flan-T5-XXL model outperforms other models in the Flan-T5 family in every evaluation metric across all SUD categories. This finding aligns with existing literature, indicating that larger models excel in zero-shot learning. Furthermore, it underscores the generalization capability of these models when presented with previously unseen data without requiring additional training. The inference is performed on a random selection of 520 annotated notes. In Table 1, for each SUD category, we report the average F1 score for the strict match and F1, precision, and recall scores for the relaxed match achieved by the Flan-T5-XXL model. Columns 2–5 show the scores calculated across the notes containing SUD information, whereas column 6 shows the same for notes containing no SUD information, and column 7 presents the combined performance scores over all notes in the test set. High scores in column 6 demonstrate that the LLM is highly capable of identifying whether there is severity information on SUD diagnostic specifiers or not.

**Table 1:** Performance scores of Flan-T5-XXL in extracting information on severity across 11 SUD categories

Substance use disorder category	Notes containing SUD information				Notes containing no SUD information	Combined
	F1 (Strict)	F1 (Relaxed)	Recall (Relaxed)	Precision (Relaxed)	F1 (Strict)	F1 (Strict)
Alcohol	61.80	83.05	87.32	84.03	93.86	82.88
Opioid	58.90	79.27	83.51	80.31	98.21	92.69
Cannabis	51.28	68.93	72.89	70.82	97.29	90.38
Sedative, hypnotic, or anxiolytic	51.61	69.33	70.18	70.98	99.59	96.73
Cocaine	54.39	79.66	85.58	80.91	92.44	88.27
Amphetamine	55.81	73.14	77.08	75.31	95.81	92.50
Caffeine	53.33	59.05	58.33	60.00	98.81	97.50
Hallucinogen	52.38	61.69	66.67	60.71	100.00	98.08
Nicotine	66.67	78.81	80.73	80.50	97.41	94.04
Inhalant	66.67	82.98	93.06	80.85	100.00	98.85
Other psychoactive substance	40.00	61.33	62.94	62.95	96.20	94.04

**Fig. 2:**
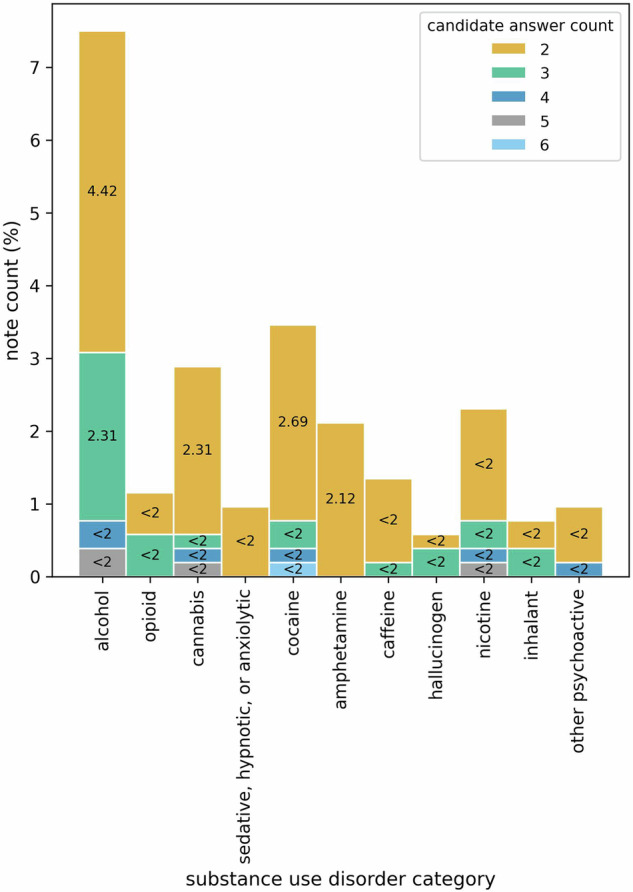
Distribution of notes with more than one candidate answer for each SUD category. Here, *x*-axis represents the SUD categories and *y*-axis quantifies the percentage of notes in the test set that have more than one candidate answer for each of the SUD categories. The stacked bars show the proportion of notes with different candidate answer counts (e.g., 2, 3, 4).

Figure 2 shows the distribution of clinical notes with more than one LLM-generated candidate answers extracting severity information on diagnostic specifiers of SUD diagnoses for 11 SUD categories. Interestingly, very few of the notes in the test set have more than one candidate answer and even fewer have more than two, as demonstrated by the percentage count of the notes. One might argue that the notes addressed in Fig. 2 may be longer than the rest, leading to an increased number of chunks and, consequently, a higher count of candidate answers. We find that for all SUD categories, there is a significantly low correlation between the length of the notes (measured by counting tokens) and the number of candidate answers—evidenced by Pearson correlation coefficients at the significance levels of 0.05, 0.01, and 0.001, as detailed in Table 2.

**Table 2:** Pearson correlation coefficient to measure the correlation between the length of the notes (in the number of tokens) and the number of candidate answers

Substance use disorder category	Pearson correlation coefficient
Alcohol	0.44***
Opioid	0.11**
Cannabis	0.26***
Sedative, hypnotic, or anxiolytic	0.12**
Cocaine	0.31***
Amphetamine	0.19***
Caffeine	0.25***
Hallucinogen	0.16***
Nicotine	0.29***
Inhalant	0.17***
Other psychoactive substance	0.24***

The significance of the correlation is measured at significance levels of 0.05*, 0.01**, and 0.001***.

**Fig. 3:**
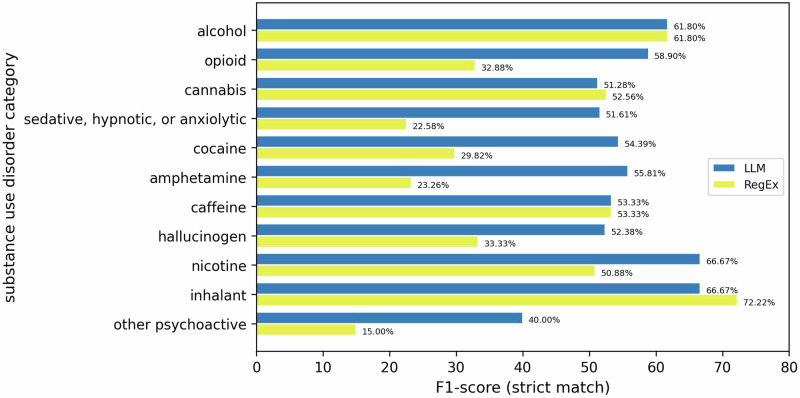
Performance comparison between LLM and RegEx across all SUD categories. Here, the *x*-axis shows the strict match F1-scores for each SUD category presented in the *y*-axis. The juxtaposed bars demonstrate the comparison between the performance of LLM and RegEx.

In Fig. 3, we compare the performance of the Flan-T5 model and RegEx across all SUD categories, evaluating F1 scores for strict matches. The findings indicate that the LLM outperforms the RegEx approach in seven out of 11 SUD categories. This difference in performance can be attributed to the nuanced and diverse expressions of SUD diagnoses in clinical notes, which do not align well with the rigid nature of RegEx. Furthermore, the better performance of RegEx over LLM in the four remaining categories (demonstrated in Fig. 3) sheds light on how some SUD diagnoses in clinical notes may be comparatively more structured than others. For instance, SUDs can be recorded as causing a secondary disorder (e.g., stimulant-induced anxiety disorder) and increase variability in diagnosis presentations. Conversely, certain substances such as caffeine, cannabis, and hallucinogens are indicated as not being able to induce certain types of secondary disorders^2^, thus reducing the variability in diagnosis presentations. Additionally, these substance categories, as well as alcohol, present less variation in the name of the substance in the diagnosis. It may be that RegEx performs better on average when there is less variation in the substance’s name, e.g., alcohol diagnoses are likely to have either “alcohol” or “ETOH”, whereas a diagnosis for opioids may have a range of parentheticals, like “opioid SUD (kratom)”, due to the variety of opioids a person may be using. Additionally, when creating the RegEx rules, we could not omit “rule out” diagnoses, i.e., diagnoses to be further assessed but not assigned to the patient at the visit. The possible presence of such notes may also affect the apparent performance of RegEx.

**Fig. 4:**
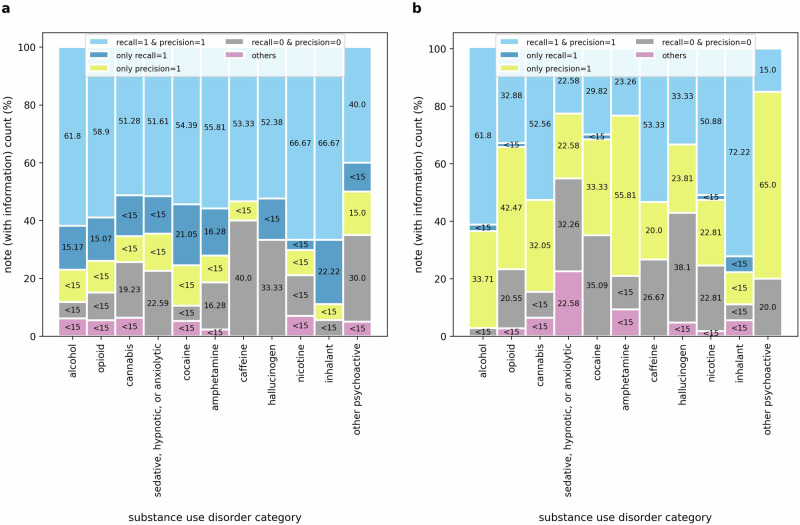
Comprehensive evaluation of the performance of LLM and RegEx. A detailed analysis of the performance of (**a**) LLM and (**b**) RegEx for notes containing any SUD information. Here, the stacked bars represent performance scores across notes containing any SUD information for five evaluation scenarios: (i) recall = 1 and precision = 1 (strict match between ground truth and prediction), (ii) only recall = 1 (complete overlap of the prediction with the ground truth), (iii) only precision = 1 (all words in the prediction are included in the ground truth; however, the reverse is not true), (iv) recall = 0 and precision = 0 (no match between ground truth and prediction), (v) others (any scenarios other than the above four).

In Fig. 4, we provide a detailed analysis of performance metrics of notes containing any mention of substance use disorders. This analysis offers a more comprehensive overview of the LLM’s efficacy in extracting information on SUD diagnosis severity. To pinpoint the nature of errors, we categorize instances based on whether the answers precisely match the ground truth or not. Specifically, we scrutinize instances where either the recall score equals 1, or the precision score equals 1, but the strict match F1-score equals 0. Additionally, we examine cases where the LLM completely fails to extract any SUD information, characterized by both recall and precision scores of 0.

A notable observation from Fig. 4a is that in a significant portion of notes where the LLM fails to strictly extract ground truth answers, it successfully extracts strings fully containing the ground truth (recall = 1). On the contrary, fewer instances with precision scores of 1 indicate that the LLM tends to generate extra words during task execution, which is an anticipated behavior. Upon observing instances where recall equals 1, it could be presumed that providing more explicit instructions, such as “Do not add any additional information in your response.”, “Extract the information verbatim from the clinical note without alterations.”, or “Restrict your response to the text in the clinical note without changing or adding any words or sentences.” could improve the strict match F1-score. However, when provided with more explicit instructions, the LLM frequently either omits critical information, such as severity specifiers or entire mentions of SUD diagnoses or introduces irrelevant details regardless, indicating challenges in striking a balance between extracting relevant content and avoiding omissions or additions. Given this trade-off, we focused on recall, reasoning that including additional context is preferable to omitting crucial information. Through iterative refinement, we determined that instructing the model to focus on SUD diagnoses and their relevant context, using prompts such as “Extract the reference to alcohol use disorder diagnosis with surrounding information relevant to it…”, resulted in relatively more accurate extraction of the target information.

Figure 4b highlights the opposite characteristics of the rule-based RegEx approach. As a more rigid string search technique, RegEx excels at capturing some parts of the ground truths—indicated by the precision scores, as opposed to finding strings containing the ground truth—depicted by the recall scores. Despite the inferior performance of RegEx compared to the LLM, further exploratory analysis reveals instances where RegEx outperforms the LLM in capturing information in clinical notes, particularly for SUD categories such as alcohol, cannabis, caffeine, and inhalants. This phenomenon could be attributed to specific note structures and should be investigated further in future studies.

**Fig. 5:**
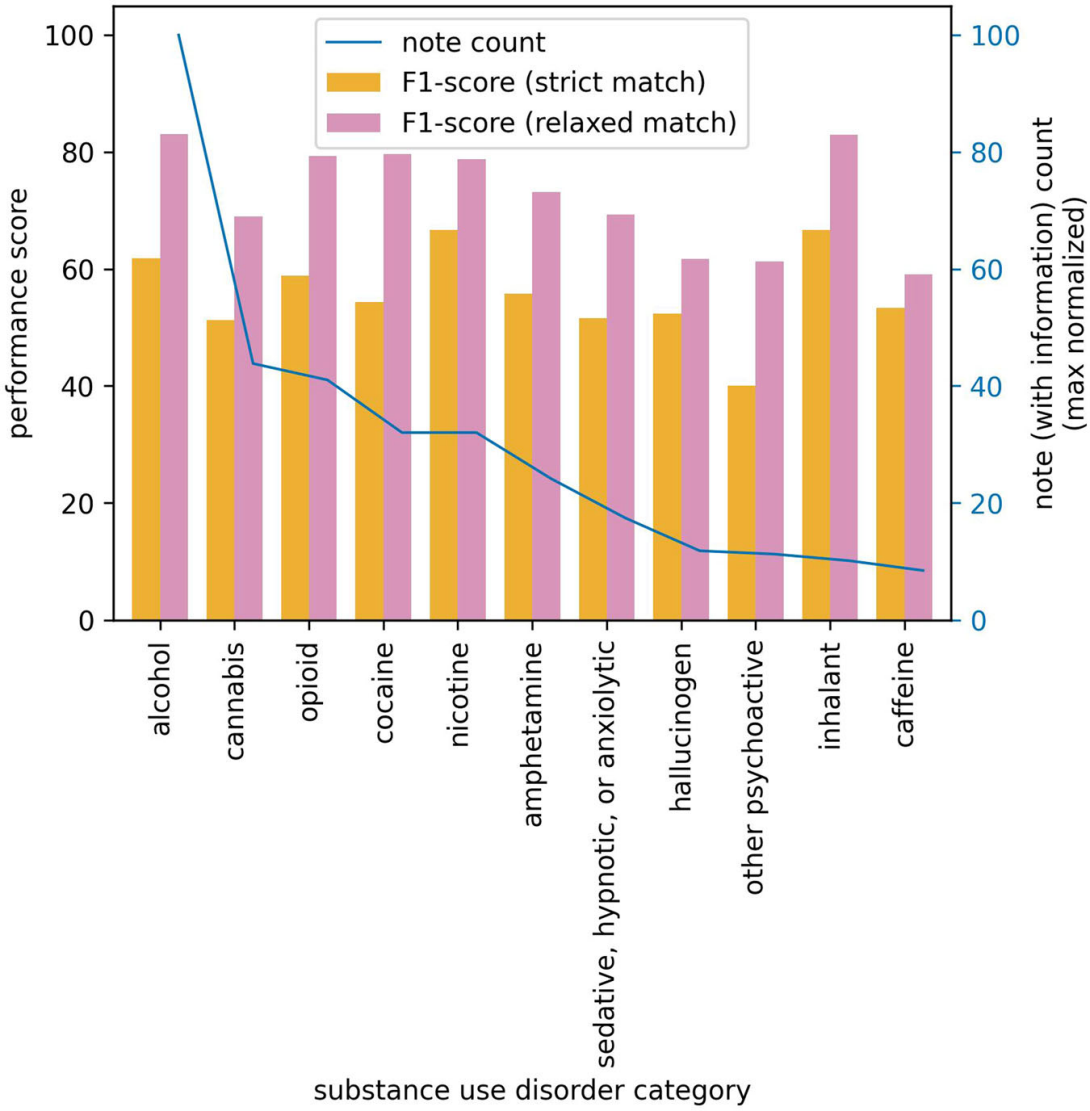
Effect of imbalanced distribution of SUD categories in the test dataset on LLM performance. The left *y*-axis represents F1 scores for strict and relaxed matches, while the right *y*-axis corresponds to the max normalized count of notes containing corresponding SUD information. The *x*-axis represents 11 SUD categories.

We investigate how the imbalanced distribution of SUD categories in the test dataset affects LLM performance. For notes that do not contain relevant SUD information corresponding to their respective categories, the LLM’s performance remains consistent across categories, as shown in Table 1. Therefore, we focus our analysis only on notes that contain specific SUD information. Despite this imbalance, as depicted in Fig. 5, our analysis reveals no distinct or consistent pattern of impact on performance, suggesting that the LLM’s performance is resilient to the uneven distribution of SUD categories.

In addition to quantitatively describing the results, we conduct a secondary error analysis to identify some probable causes of discrepancies between the LLM responses and clinician-set ground truths. We manually review a sample of 55 cases, selecting five examples from each SUD category where the LLM outputs diverged from the SME, and discuss these discrepancies with the SME. We find instances where the LLM misinterprets SUD mentions in the past problem list as diagnoses. For instance, a note may state “… previous encounters problem list includes: \n {X} cannabis abuse \n {X} alcoholism or alcohol abuse”. The LLM sometimes misinterprets such cases as diagnoses although they are clearly mentioned to be past problems. We also observe hallucination errors related to severity specifiers of SUD diagnoses, particularly when multiple SUD diagnoses with different severity levels were documented together in the clinical notes. For example, the note mentions “alcohol use disorder, unspecified \n severe cocaine use disorder” but the LLM responds with “severe alcohol use disorder”. Errors also occurred when the LLM failed to recognize or accurately identify SUD diagnoses that were present in the clinical notes. One notable pattern was the incorrect identification of “stimulant use disorder” as “amphetamine use disorder,” whereas the SME determined that the stimulant in question was cocaine based on contextual cues and prior clinical knowledge. For stimulants such as cocaine, amphetamine, and caffeine, such errors likely stem from imprecise coding standards in ICD-10 stimulant use disorder, which occasionally clinicians write in clinical notes as opposed to more granular DSM-5 diagnoses. For missed information, we identified instances where the LLM failed to detect any SUD information and responded with “unanswerable”. These cases did not exhibit any discernible patterns of failure, underscoring the need for further in-depth analysis to better understand the underlying causes of these errors. We also observe that the LLM occasionally provides more detailed responses compared to the SME-established ground truth. For example, while the ground truth may be “alcohol use disorder, mild”, the LLM elaborates with “alcohol use disorder, mild r/o unspecified neurocognitive disorder (per record)”. Similarly, for a ground truth of “severe cocaine use disorder”, the LLM expands with “veteran appears to meet DSM-5 criteria for severe cocaine use disorder based on the self-reported symptoms: …”. We also notice that the LLM sometimes includes other SUD diagnoses along with the one that has been prompted, especially when different SUD diagnoses are written together. For example, “inhalant use disorder, severe; opioid (kratom) use disorder, severity unspecified; and stimulant use”. While this demonstrates the model’s capability to enhance its responses with additional clinical context when available, it poses challenges for interpreting the response and creating structured data from the LLM-generated text. Overall, this analysis highlights the common error types and provides insights into specific challenges faced by the LLM in accurately identifying SUD severity-related information in clinical notes, emphasizing areas for model improvement and targeted adjustments.

In evaluating the practicality of applying LLMs versus rule-based algorithms to large datasets, it is crucial to consider not only the accuracy of these approaches but also their computational and development demands. Extracting information from the test set of 520 clinical notes of varying lengths using Flan-T5-XXL takes approximately 34 minutes of GPU compute time per SUD category, with a batch size of 1. On the other hand, the compute time for RegEx to extract information from the test set of 520 clinical notes of varying lengths per SUD category is approximately 0.02 min. However, developing and maintaining a rule-based system, such as one using RegEx, involves considerable manual effort. Creating and fine-tuning RegEx patterns requires substantial time and input from SMEs, which is hard to quantify. Furthermore, these patterns need periodic updates to capture evolving language use, and even with these efforts, there is no guarantee of capturing all relevant language variations effectively. Therefore, while the LLM might require more computational resources, it offers the advantage of lower ongoing development effort and can handle large datasets more efficiently. A rule-based system, on the other hand, though potentially less computationally demanding, demands significant manual development and maintenance effort. This trade-off should be considered when evaluating the feasibility and benefits of each approach for processing large datasets.

## Discussion

LLMs offer significant promise in enhancing clinical practice by facilitating the extraction of crucial information from lengthy and intricate clinical documents. Unlike conventional deep learning models, LLMs often do not necessitate supplementary training to adjust to novel tasks, thus alleviating the bottleneck of annotating large quantities of high-quality data for new downstream tasks. In this study, we address the challenge of determining severity-related SUD diagnostic specifiers from clinical notes—a daunting task owing to the variations in how they are written in clinical notes. Examples of this variation that indicate the difficulty in using strictly rule-based methods (including variations in spelling, abbreviations, and syntax) are:

- cannabis/alcohol/opioid use disorder: mild
- alcohol use disorder mod/severe
- moderate caffeine use do
- opioid (heroin/ vicodin) use disorder - severe (on agonist therapy)
- marijuana user (in remission)
- cannabis (thc vape) use disorder, mild
- (ephedrine) sedative use disorder, in sustained remission
- sedative hypnotic use disorder, severe (xanax)
- meets criteria for substance use disorder: cocaine [] mild (2-3); [] moderate (4-5); [x] severe (6 or more)
- amphetamine (methamphetamine) or other stimulant, without perceptual disturbances disorder, sever, in remission
- other hallucinogen use disorder (mdma/ecstasy), moderate, in remission
- other/unknown substance disorder: severe (coricidin)
- moderate inhalant (nitrous oxide) use d/o

This study is the first foundational effort in a broader goal to extract additional granular risk factors associated with patients with SUD. These include but are not limited to severity-related SUD diagnostic specifiers, chronology, co-determinants (e.g., withdrawal symptoms), and social determinants of health—often excluded from the structured data in the electronic health records. This detailed information plays a vital role in risk assessment, guiding effective treatment plans, ensuring patient safety, and fostering recovery and overall well-being. We began this effort with severity-related SUD diagnostic specifiers in the clinical notes as a proof of concept in part because such specifiers are clearly indicated and described in the DSM-5 (e.g., mild, moderate, and severe)^2^. Yet, even with such clear documentation for these specifiers, our work has shown that additional research is necessary with both the LLM and rule-based approaches to achieve high levels of accuracy. In this vein, other risk factors that are less clearly documented (social determinants, for instance) are likely to pose additional challenges. To the best of our knowledge, no prior studies have specifically targeted the extraction of diagnostic specifiers across 11 distinct categories of SUD diagnoses from clinical notes.

Our findings indicate that, when provided with appropriate prompts, the Flan-T5 model excels in extracting information with high recall and precision across most SUD categories. Particularly, it outperforms RegEx in extracting nuanced information that cannot be easily captured by rigid rules. Nonetheless, the LLM occasionally struggles to extract information accurately and succinctly, suggesting that an ensemble approach combining both RegEx and LLM may be beneficial in such instances. Moreover, the errors observed in the study indicate that finetuning the LLM based on specific instructions could enhance overall performance.

There are several key limitations in this study. Firstly, the evaluation was conducted on a relatively small test dataset comprising 520 annotated notes, with even fewer containing the target information. Expanding this annotated dataset necessitates significant manual effort from SMEs. As a future direction, we aim to explore more efficient annotation methods, potentially using a hybrid approach that combines LLMs and RegEx to assist SMEs. Secondly, our study exclusively utilized Flan-T5 models. Future research could benefit from experimenting with LLMs of diverse architectures, pre-training paradigms, pre-training datasets, and higher model parameters, such as LLAMA2 or LLAMA3. Also, we could not use closed models such as GPT-4 due to privacy concerns and data-use restrictions. Thirdly, exploring instruction-finetuning to enhance the LLM’s performance for this specific task is subjected to further efforts. Additionally, it is important to acknowledge that Flan-T5 models, similar to their counterparts, are susceptible to hallucinations and bias. While our post-processing methods aim to mitigate hallucinations to some extent, careful consideration and attention are essential when considering the utilization of such models in critical and sensitive clinical applications.

## Data Availability

The dataset developed for this study is not accessible to the public under requirements of the Health Insurance Portability and Accountability Act of 1996 and related privacy and security concerns. The underlying electronic health record data can only be used for improving treatment for patients receiving services from the Veterans Health Administration (VHA). Those interested in accessing VHA EHR data extracts curated for this quality improvement project to replicate and validate findings may contact the corresponding author regarding access via VHA collaboration.
